# Optimal Learning Paths in Information Networks

**DOI:** 10.1038/srep10286

**Published:** 2015-06-01

**Authors:** G. C. Rodi, V. Loreto, V. D. P. Servedio, F. Tria

**Affiliations:** 1 Polytechnic University of Turin, Dept. of Mathematical Sciences, Corso Duca degli Abruzzi, 24, 10129 Turin, Italy; 2 Institute for Scientific Interchange (ISI), Via Alassio 11C, 10126 Turin, Italy; 3 Sapienza University of Rome, Physics Dept., Piazzale Aldo Moro 2, 00185 Rome, Italy; 4 SONY-Computer Science Lab (CSL), 5, Rue Amyot, 75005, Paris, France; 5 Institute for Complex Systems (ISC-CNR), Via dei Taurini 19, 00185 Rome, Italy

## Abstract

Each sphere of knowledge and information could be depicted as a complex mesh of correlated items. By properly exploiting these connections, innovative and more efficient navigation strategies could be defined, possibly leading to a faster learning process and an enduring retention of information. In this work we investigate how the topological structure embedding the items to be learned can affect the efficiency of the learning dynamics. To this end we introduce a general class of algorithms that simulate the exploration of knowledge/information networks standing on well-established findings on educational scheduling, namely the spacing and lag effects. While constructing their learning schedules, individuals move along connections, periodically revisiting some concepts, and sometimes jumping on very distant ones. In order to investigate the effect of networked information structures on the proposed learning dynamics we focused both on synthetic and real-world graphs such as subsections of Wikipedia and word-association graphs. We highlight the existence of optimal topological structures for the simulated learning dynamics whose efficiency is affected by the balance between hubs and the least connected items. Interestingly, the real-world graphs we considered lead naturally to almost optimal learning performances.

Modern global positioning systems allow human beings to locate themselves and find their way in the physical space with an unprecedented accuracy. GPS technologies beautifully complemented space perception humans naturally possess. The idea of moving on a space is actually far more general and we, as humans, constantly wander in what could be defined an information or a knowledge space, i.e., a complex structure linking, through semantic and logic relations, pieces of our knowledge and culture. Nowadays, the notion of knowledge or information space is not only an abstraction and information networks are widespread, from the whole World Wide Web to the paramount example of Wikipedia^1^,^2^,^3^, from word-association graphs^4^,^5^ to ontologies and taxonomies. Whenever we work, study, play, we naturally and constantly navigate information networks and our activities could be intuitively thought as a path on a network of points encoding information and knowledge. But how we stand and how we shape our way in this space as well as the structure of this space itself are often largely unknown. In this sense, nowadays, the Socratic “know thyself” is far from being a concrete reality. Still a better knowledge of our trajectories in knowledge spaces would be key to better design learning, professional or leisure activities.

The explosion since 2012 of Massive Open Online Courses (MOOCs) witnesses the exponential growth in the demand for access to education. The recent success of web platforms and applications designed for learning, e.g., Anki^6^ or Duolingo^7^, reveals an increasing interest in educational software, which could provide self-learners with tailored, efficient and innovative tools for learning. Within this framework, the research of optimal educational algorithms has to deal with the problem of finding the best *scheduling* of the study practices, i.e., the best timing for introducing new material and reviewing the older, in order to make the retention enduring and to minimize the forgetting. In a recent pioneering work^8^, Novikoff *et al.* beautifully formalized mathematically this problem and developed some models for the generation of learning schedules that would yield to lifelong learning or cramming, without any forgetting during the time considered.

In the scheme proposed in^8^, the knowledge to be acquired is pictured as a set of independent units. To make a step towards a more realistic scenario, correlations among bits of information must be also considered. Our work moves along this direction, by using a complex network representation of the units to be acquired and their interconnections.

The complex systems perspective in dealing with cognitive and linguistic systems is not novel in the literature. Indeed, the first efforts to depict the semantic memory through graphs of concepts go back to the sixties, with the Quillian’s model of the semantic memory and its successive generalizations^9^,^10^. More recently, the complex network approach has become widely used, for instance to gain a deeper understanding and characterization of the properties of semantic networks^5^,^11^ and possibly even to model the mechanisms underlying their growth^12^. Furthermore, also the dynamics of cognitive processes have been addressed and investigated within the framework of the network theory as diffusion processes on the networks. Successful examples of such approach regard the extraction of semantic similarities relations on graphs of free-associated words^13^ or the predictive analysis of fluency tasks by means of algorithms like the PageRank^14^. The reviews by Borge-Holthoefer and Arenas^15^ and by Baronchelli *et al.*^16^ provide a comprehensive overview of the complex network contributions to the investigation of language and cognitive science.

Here the cognitive task we model is a learning process of a-priori defined collection of items embedded in a complex network. In particular, we focus on the role that the structure of this network, i.e., the set of logic or semantic links among the different bits of knowledge, can have in enhancing or hindering the acquisition and retention of information, thus determining the learning efficiency. In this work we consider both synthetic graph structures and real-world ones, namely some subsections of the Wikipedia graph and the Human Brain Cloud network of free word associations^4^, both of them taken as proxies of information and knowledge spaces.

In order to investigate how the topology and the statistical properties of the posited complex network structures can affect the efficiency of the learning process, we introduce a general class of algorithms for the generation of a *learning schedule*, i.e., an ordered sequence of item presentations. In other words our algorithms generate paths in the network structure, where a path is defined as an ordered set of visits to the nodes of the network. The whole process implies a subtle balance between the introduction of new units and the repetition of old ones, also taking into account the possibility of failures of the learning procedure, i.e., forgetting episodes, and the corresponding retrieval processes.

As in the work cited^8^, our starting point are some results of the century-long cognitive science research on cognition and memory. In particular, we focus on how the allocation over time of the study practices for each item can affect the learning performance. In his 1885 milestone work^17^, Ebbinghaus introduced the *spacing effect*. This finding refers to the notion that spreading the study sessions of any item over time makes its retention more durable than massing them in a short period, where the inter-study session intervals can be empty or filled with practices of other items. Many references can be found in literature on both the theoretical discussion of the psychological mechanisms involved^18^,^19^ and on some experimental evidences of the validity of this effect^20^. Furthermore, among all the possible inter-study intervals, it has been reported^21^,^22^,^23^ that the benefits gained by spacing are enhanced if, for each item, the intervals between its study practices expand with the reviews rather than remaining fixed. This phenomenon is usually referred to with *lag effect* or *expanded retrieval*.

Standing on the shoulders of this copious literature, our class of learning algorithms incorporate the above mentioned effects, namely the spacing and lag effects, in the generation of learning paths. We also make assumptions on the role that connections between items may play in the schedule planning. We suppose that while learning, semantically related concepts could be primed or reinforced in memory, thus adapting to a learning process some of the suggestions of the seminal spreading-activation theory for information retrieval^9^,^10^. Further fundamental references are some results of previous research on the early words learning in toddlers^24^,^25^ or in second language learners, for which cognitive rather than linguistic associations seem to enhance the acquisition process^26^.

Our main result is that the acquisition process is strongly affected by the topology of the underlying knowledge network. We observe that a notion of optimality in the learning process can be introduced and that optimal performances can be obtained if the underlying graph features small-world and scale-free properties with a balance between the number of hubs and of the least connected items. Surprisingly the real-world networks we analyzed here turn out to be close to optimality. That is the case of the networks based on collaborative tasks or spontaneous activity of users, like some subsections of Wikipedia and the Human Brain Cloud dataset of free-associated words, both considered in our work. This finding represents a very interesting hint towards a subtle link between the way in which humans construct knowledge spaces and the way in which they possibly explore them, retrieve the information and learn.

## Results

### The model

**Figure 1:**
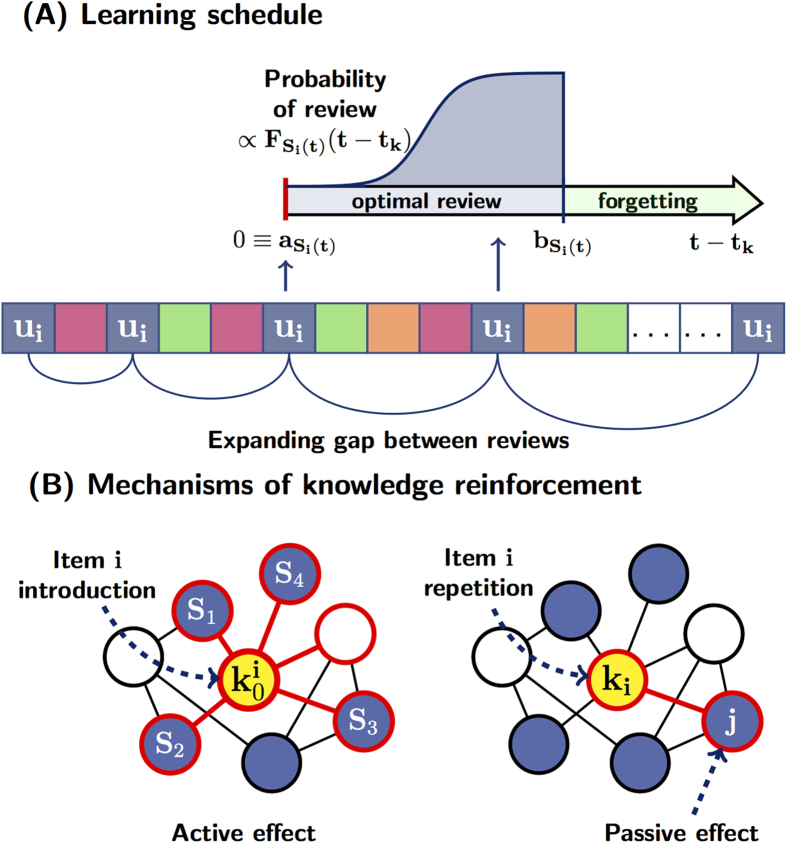
Model illustration. (**A**) In a learning schedule the interval required between any two successive presentations of the same item *i* expands with the number of reviews. To this end the probability of a repetition is computed for every node already introduced as illustrated. If the *k*-th presentation of a node *i* with knowledge strength *S*_*i*_ (see the main text) occurred at time *t*_*k*_, the (*k* + 1)-th happens at time t with probability proportional to 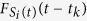. This function is non null in the temporal interval 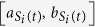, whose bounds are increasing functions of the total knowledge strength of item *i*. After 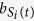 steps without being repeated, the item *i* is forgotten and has to be reintroduced. In (**B**) the supposed mechanisms of knowledge reinforcement are illustrated. When item *i* is introduced, it gains a starting knowledge value 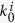 depending on how much its neighborhood is known. This mechanism is referred to as *active effect*. Afterwards, at every successive repetition, its knowledge is reinforced by 1 and one among its introduced neighbors, say *j*, is randomly selected to receive a *passive* reinforcement, i.e., 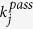 is incremented by a quantity *α*. In all our simulations *α* = 0.1.

We represent the set of items to be learned as nodes in a graph and we model learning as a dynamical process through which we construct a *learning schedule*, defined as a sequence of successive visits an hypothetical student would make to the nodes of the graph. At each extraction either a new node (never visited before) can enter in the sequence, or an already considered one can be repeated (subfigure (A) of Fig. 1). In particular, at each time step, the item *i* to be presented to the student, i.e., appended to the learning sequence, is stochastically chosen according to three factors: (i) the time, *t*_*i*_, elapsed for each item *i*, since its last presentation; (ii) the time, *t*_*new*_, elapsed since the last introduction of a brand new item; (iii) the knowledge strength *S*_*i*_(*t*) of item *i* at time *t*. The algorithm takes into account both the number of times the item *i* has already been repeated, and the repetitions of items connected to it in the graph, that is the knowledge of the context of *i*. In particular, the knowledge strength is the sum of three distinct contributions (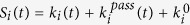), corresponding to three different mechanisms that are supposed to lead to the acquisition and reinforcement of any item knowledge: (a) *k*_*i*_(*t*) is the number of time the item *i* is repeated since its first introduction or since its reintroduction from the forgetting queue (see next paragraph for its definition); (b) 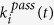: every time an item is repeated, one among its neighbors already introduced (and not forgotten), say *i*, is randomly selected (uniformly or with probability proportional to the weight of the connecting link, respectively in unweighted or weighted graph) and 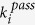 increases by a value *α* < 1. We name this process *passive effect*; (c) when an item *i* enters in the sequence for the first time or from the forgetting queue, its starting knowledge 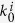 is a weighted average of the knowledge acquired so far on its neighbors. We name this the *active effect* and we refer the reader to the methods section for its complete definition.

Constraints on the time window useful for reviewing an item are provided, implementing the spacing and lag effects. As in a previous work^8^, two successive occurrences of the same item *i* should occur inside a given interval 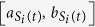, whose bounds are monotonic non-decreasing function of the knowledge strength *S*_*i*_, in order to prevent the forgetting of the item. Our agenda generation rule is thus the following. At each discrete time *t*, for each item *i* among the *n*(*t*) already introduced in the schedule, the temporal distance since its last occurrence is evaluated: Δ_*i*_*t* = (*t* − *t*_*i*_), where *t*_*i*_ is *t*he last time at which the item *i* entered in the sequence. If 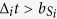, the item is forgotten, put into a *forgetting queue* and its knowledge strength *S*_*i*_ is reset to zero. If 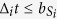, a monotonic non-decreasing function of Δ_*i*_*t*, 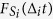, de*t*ermines the probability for node *i* to be repeated at time *t* (refer to the methods section for its definition). The probability of introducing in the sequence a new item instead of repeating an already introduced one depends linearly on the time elapsed since the last introduction of a novel item (we refer again the reader to the method section for a complete definition of the probabilities). In the case of a new introduction event, the oldest item stored in the forgetting queue is reintroduced, without updating *t*_*new*_. If the forgetting queue is empty, a brand new node is introduced to the learning schedule and *t*_*new*_ is updated.

We investigate different criteria determining the particular brand new node to be introduced in the learning schedule in order to investigate the effect of the semantic structure underlying the items to be learned (other possible criteria are presented in the Supplementary Information): (i) random learning (RL): each new entry is randomly and uniformly selected among the ones not already presented; (ii) preferential acquisition (PA): the new entries are chosen with probability proportional to their degrees (or strength, in case of weighted graph). In doing so, we reproduce the preferential acquisition model for the early words learning in toddlers discussed by Hills *et al.*^25^; (iii) random surfing (RS): every time a new item has to be chosen, with probability *p* a nearest neighbor not already introduced of the last item introduced in the sequence is selected, if any, with probability proportional to its degree. Otherwise, with probability (1 − *p*) or in case all neighbors were already introduced, a jump is made in the network and a random node is selected with a PA step. In case of weighted graph, strengths are considered instead of degrees. This criterion is reminiscent of the PageRank algorithm^27^.

### Outcomes

On the generated sequences, two main quantities are studied to evaluate the efficiency of the corresponding learning processes. The first one is the introduction rate *n*(*t*), namely the number of distinct nodes presented throughout the sequence as a function of time and not forgotten. The second variable is the graph coverage time, that is *t*_*N*_ such that *n*(*t*_*N*_) = *N*, i.e., the time needed to present every node at least once and to empty the forgetting queue. For these quantities two different behaviors can be expected in the limit cases of totally disconnected and connected graphs. Because of the generation rule previously explained, and in particular the active knowledge reinforcement term, interconnections between nodes lead to a faster rate of introductions and therefore to a shorter coverage time. However, for intermediate connectivity values, the learning efficiency does depend on both the topology of the graph explored and, for a given topology, on the criterion according to which novel nodes are to be introduced. For this, we carried out simulations on different types of synthetic graphs and on networks generated from real data. In the first case, for each graph type, we have compared sequences obtained from graphs with increasing average degree. For the real networks, methods of perturbation have been developed to increase and decrease the connectivity while only slightly modifying the other statistical properties, such as the degree or strength distributions. They are described in the Methods section.

**Figure 2:**
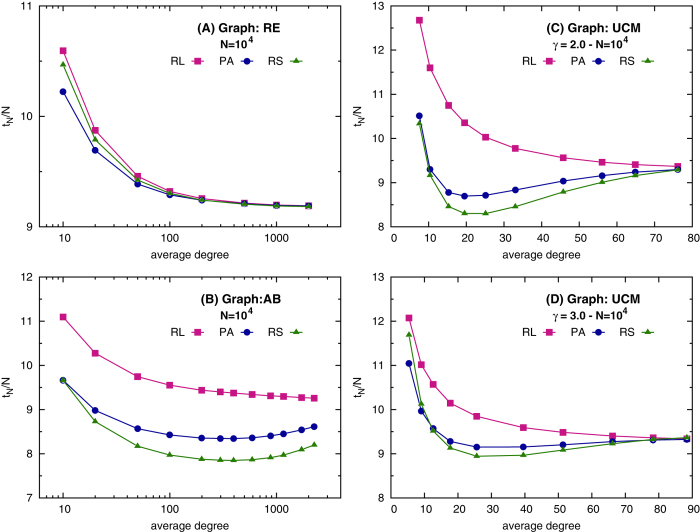
Coverage times on synthetic graphs. In the figures, the mean coverage times scaled to the network order *N* = 10^4^ as a function of the network average degrees are shown for different synthetic graphs, generated according to the (**A**) Erdös-Rényi model^28^, (**B**) Barabási-Albert model^29^, (**C**) uncorrelated configuration model^30^ where *P*(*deg*) ∝ *deg*^−*γ*^ and *γ* = 2, (**D**) same as in (**C**) with *γ* = 3. The data are averaged over 10 different graph realizations and 5 learning agendas for each of them. Standard errors are also reported but they are covered by symbols. Different colors refer to the three criteria used to select the entries: random learning (RL, magenta), preferential acquisition (PA, blue) and random surfing (RS, green). Note the logarithmic scale of the horizontal axis for (**A**) and (**B**).

**Figure 3:**
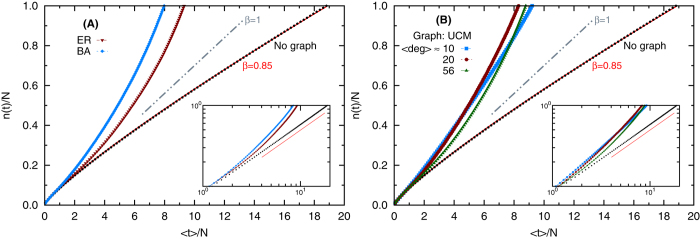
Introduction rate on synthetic graphs. Fraction of distinct nodes introduced as a function of the (rescaled) average time needed to cover them. In figure (**A**) we report data obtained on Erdös-Rényi model^28^ and Barabási-Albert model^29^ graphs with average connectivity 〈*deg*〉 ~ 100. In (**B**) the graphs considered are generated according to the uncorrelated configuration model^30^ with *P*(*deg*) ∝ *deg*^−*γ*^ and *γ* = 2 and different average connectivities. In all the cases, the graph size is *N* = 10^4^ and the algorithm used to select the entries is the random surfing RS. In both (**A**) and (**B**), with black dots we report the introduction rate for an equivalent set of uncorrelated items. It is fitted with a sub-linear curve *y* ∝ *x*^*β*^, with *β* = 0.85. The fitting curve is shown with red line in the main graph, while in the insets we report an eye-guide power-law with same exponent. An eye-guide linear (*β* = 1, grey line) curve is also reported in the main figures. The insets axes are in log-log scale. The data are averaged over 50 agenda simulations. Standard errors are reported, though not visible at the plot scale.

A global insight into the role of the graph topology and its connectivity properties can be gained by comparing the coverage times. In Fig. 2 we report the main coverage time obtained for four different synthetic network types, both random graphs^28^ (subfigure (A)), and different scale-free graphs: BA^29^ (subfigures (B)) and graphs generated with the Uncorrelated Configuration Model (UCM)^30^ (subfigures (C)-(D)), and for the three entry selection criteria earlier defined. Scale-free graphs together with no random criteria of exploration lead to optimal learning performances for intermediate average connectivities. The improvement in the coverage time is even more meaningful in graphs with the same maximum degree but a larger fraction of hubs, as it emerges by comparing the UCM networks with two different exponents of the degree probability distribution, reported in subfigures (C) and (D). With regard to the selection criteria, an efficiency gain is achieved in the scale-free graphs when they are locally explored, namely when the random surfing criterion is used. A greater insight into the dynamics of the learning schedule construction process is given by looking at the introduction rate *n*(*t*). In Fig. 3 (subfigure (A)) results on random^28^ and BA^29^ graphs with similar average degree are compared. In subfigure (B) the data refer to graphs generated with the UCM model with low, intermediate and high values of average connectivity, and exponent *γ* = 2 in the power law degree distribution *P*(*deg*) ∝ *deg*^−*γ*^. In both figures, we contrast the data with the results obtained on an equivalent set of completely disconnected nodes (and a linear trend is also reported for comparison). For uncorrelated items, the introduction rate turns out to be a sub-linear function of time (*n*(*t*) ≃ *t*^*β*^, with *β* < 1), in accordance with Heaps’ law^31^. Instead, for items embedded in a graph, two different behaviors can be identified. As long as the graph is largely unexplored, the introduction rate has the same trend as in the case of disconnected items, namely sub-linear. Later on along the learning dynamics, new items are introduced with higher frequency, featuring a super-linear tail for the introduction rate, i.e., *n*(*t*) = c^*^t^*γ*^, with *γ* > 1 and c^*^ << 1. Note that, for a short time interval, such a super-linear rate is still compatible with the schedule constraint that at most one brand new unit can be introduced at each discrete time. The origin of this super-linear behaviour is related to the active effect contributing to the knowledge strength of each item (see the model subsection and the methods section). Indeed, when a significant fraction of items have already been introduced, new items typically enter the schedule with higher and higher knowledge strengths, thus requesting longer intervals before they need to be reviewed, allowing in this way the introduction of further new items.

**Figure 4:**
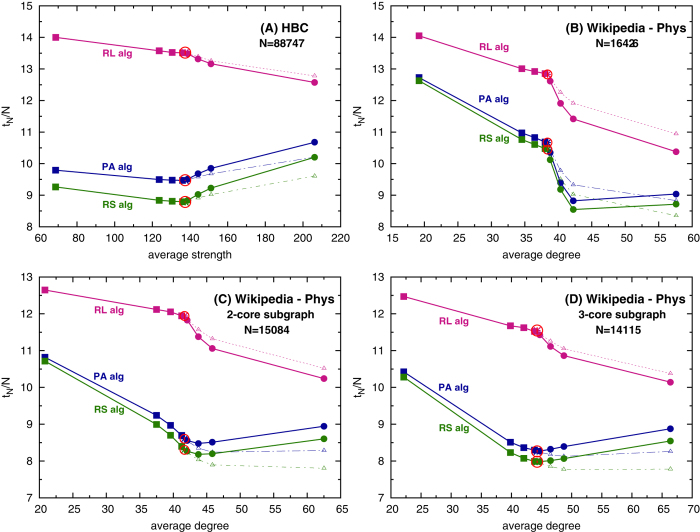
Coverage times on real-world graphs. It is reported the coverage times obtained by simulating the learning agendas on a real-world graph (red circled points) and on three perturbed versions of it. The real-world graphs are: (**A**) the weighted network generated from the Human Brain Cloud^4^ dataset, (**B**) the Physics section of Wikipedia and its (**C**) 2-core and (**D**) 3-core subgraphs. In each figure, the squares refer to data resulting on graphs with reduced connectivity, obtained by randomly selecting and deleting different amounts of links in the original graphs. With circles and triangles data are reported when two different procedures for increasing the connectivity are considered. In the first case (circles, solid line), links are created by randomly selecting pairs of unconnected nodes. In the latter (triangles, dashed line), new links are added only between second-neighbor nodes. In all the cases, the fraction of links deleted/created are equal to 0.01, 0.05, 0.1 and 0.5. Different colors refer to the three criteria used to select the entries: random learning (RL, magenta), preferential acquisition (PA, blue) and random surfing (RS, green). The data referring to the unperturbed graphs are averaged over 50 agendas. In all the other cases, for each type of perturbation procedure and percentage of edges added or removed, 10 different perturbed versions of the graphs are generated and 5 agendas are simulated on each of them. Then, the averages are done over the 50 aggregated agendas realizations. Standard errors are reported, though not visible at the plot scale.

The coverage times resulting from simulations on real-world graphs and their perturbed versions are shown in Fig. 4. Data in subfigure (A) refer to weighted, undirected graph generated from the Human Brain Cloud^4^ word association dataset. In particular, a filtered version of the data was provided by Gravino *et al.*^5^. For the data reported in the other subfigures, we considered the subgraph in Wikipedia^32^ corresponding to the Physics subsection. The procedure implemented to extract it is reported in the Methods section, while some statistical properties of the graphs considered are analyzed in the SI.

As for the synthetic graphs, the random learning algorithm for choosing the new entries does not lead to meaningful performances, the coverage time monotonically decreasing as the connectivity enlarges. On the contrary, when the information stored in the topology is used to more shrewdly select the novel nodes, the minimal coverage time is achieved for intermediate connectivities. Moreover, the structures leading to the optimal performance coincide with the original HBC graph (subfigure (A)) and with the original Physics graph, when the least connected nodes are removed, i.e., when the inner cores are considered and treated as unperturbed new graphs. In particular, the results obtained on the Physics original graphs (subfigure (B)) and its more external inner cores (the 2-core in subfigure (C) and the 3-core in (D)), suggest a meaningful role of the poorest connected nodes in affecting the learning efficiency. Indeed, as soon as the leaves are removed, the topology of the Wiki subgraph becomes closer to the optimal one, with respect to a further increase of the number of connections. This finding can be used in future to suggest a topological reorganization of Wikipedia subgraphs resulting in an optimization of thematic learning paths. By looking at the data acquired when the two positive perturbation procedures are implemented, it can be concluded that it is not the average connectivity that triggers the most efficient learning performance, rather the relative presence of poorly connected nodes with respect to the hubs.

## Discussion and Conclusions

In this paper we investigated the role of the topology of complex information and knowledge networks when generating efficient learning schedules for the items they embed. We proposed a general class of stochastic algorithms to sequence the introductions of the different items and their reviews over time, while satisfying some constraints on the best timing, as they can be derived from previous results of cognitive science research. Furthermore, we studied how the topological structure representing the complex semantic and logic relationships among the items to be learned can affect the learning procedure. We investigated, in particular, how different statistical properties and topologies of the graphs in which the items are embedded affect the process, as well as the ways such graphs should be explored while introducing new material in order to achieve efficient learning paths.

Our results show that some topologies lead to optimal learning schedules, i.e., schedules that minimize the learning time while preventing forgetting episodes. They are small-world, scale-free structures, in which the relative number of hubs and low-connected nodes are balanced. In fact, structures with either too many hubs or poorly connected nodes hinder the learning process. In the first case, the context for items is indeed too large to take advantage of it. In the latter case, the more specific and low connected the nodes, the more difficult it is to access them or to achieve a gain in the knowledge reinforcement throughout the learning process. Furthermore, we find that the order through which the networks are explored as new items are introduced in the agenda is essential for taking full advantage of the topology features, a random exploration turning out to be ineffective in eliciting the information stored in the graph.

Finally, a very interesting outcome of our study is that the real-world graphs we considered here, the Human Brain Cloud word-association network and the Wikipedia graph, turned out to be almost optimal with respect to the criterion described above. This points to a subtle link between the way in which humans organise their knowledge, i.e., the structure of the knowledge space, and the way in which the information could be retrieved, for instance through a learning path. From a technological perspective this is very interesting since it suggests the existence of a feedback loop between the dynamical evolution of information networks, i.e., the way in which users shape them by contributing content and semantics, and the way in which users navigate the sea of information and knowledge. This can lead to an improvement of both editing and navigation strategies and suggests: (i) both a brand new role for users and editors in information networks, e.g., not only content provider but more and more crucially path designers, and (ii) a leading direction towards search engines for learning paths.

In summary, the outcomes presented here suggest a key role of the conceptual structures embedding the items to be learned in making learning processes faster and the retention longer. From this perspective, empirical research on how different patterns of associations could drive the acquisition of new concepts would be key to progress and proceed towards more informed algorithms to generate learning paths. This understanding can help in designing novel educational software and more in general to improve both the teaching ability of mentors and the learning experiences of both students and individual self-learners. We believe this approach can have a far-reaching impact since a better understanding of the complexity of our knowledge spaces, as well as the way in which we navigate them, may have the potential to trigger the development of new tools to orient human beings in complex information networks to better shape education, professional growth and leisure activities.

## Methods

### Definition of the active effect 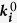


The starting knowledge 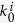 of an item reflects the knowledge of its context, i.e., of its neighbors. It is defined by:
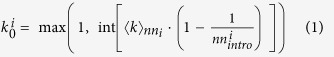
where 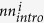 is the number of neighbors of *i* already introduced and not forgotten and 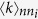 is the average no-passive knowledge strength over the set of neighbors 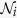 of item *i*. It is defined as:
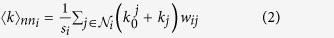
where *w*_*ij*_ is the weight of the link connecting node *i* to node *j* (*w*_*ij*_ = 1 in an unweighted graph) and *s*_*i*_ is the strength of node *i*: 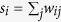.

### Probabilities of selecting a node *i*

An item *i* is chosen to be repeated or a new one is introduced according respectively to the following normalized probabilities:
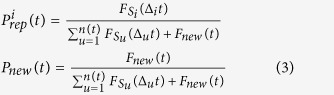
where *F*_*new*_(*t*) = 1/2 (*t* − *t*_*new*_), being *t*_*new*_ the time of the last introduction of a brand new item in the learning sequence.

### Definition of the function 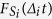


Here we choose to set 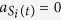 and 
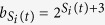
, as illustrated in Fig. 1, subfig. (A). In so doing, we suppose that the temporal window useful for a review to occur expands exponentially with the number of reviews^33^. Moreover, in order to take full advantage of the lag effect, we choose the repetition probability function so that a review is more likely to happen the closer the time is to the upper bound 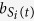. For each item *i*, we define 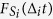 as
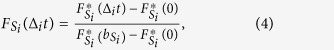
where
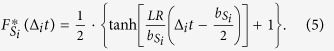
In this definition, we introduce *LR* as the only free parameter, which stands for *learning rigidity* and fixes the function slope. In the following, we consider *LR* = 2^3^, while tests are reported in the Supplementary Information on how its value affects the learning efficiency.

### Perturbation of real-world graphs

Starting from a real-data based graph, a predefined percentage of links were created or deleted according to the following criteria. When it was required to remove some connections, they were randomly selected and deleted, regardless of their weights or of the degrees of the connected nodes. As a main consequence, some disconnected components might emerge. In adding links, two different strategies were implemented. In a first case, two reciprocally disconnected nodes were randomly selected and a connection was created between them, no matter their distance on the graph. According to a second procedure, a node was randomly selected and a new connection was created with one among its second-neighbors. In both cases, the new link weight was possibly assigned by sampling the original weight distribution. In particular, an edge in the original network was randomly selected, and its same weight was assigned to the new link. How the perturbation procedures affect the graphs strength or degree distribution is reported in the Supplementary Information.

### HBC data filtering procedure

The data set here considered is a modified, undirected filtered version of the original one, provided by Gravino *et al.*^5^. Details on the filtering procedure are reported in the Appendix A of their work.

### Wikipedia subsection extraction

The MediaWiki API were used^34^. First, the list of thematic Wikipedia article titles was fetched by enquiring the API for the corresponding scientific area, i.e., by restricting to the corresponding category, e.g., *Category: Physics articles by importance*. Then, each page referring to the titles collected was scanned for the included links to other pages. Pages containing talks, templates and categories were not taken into account as well as connections toward pages not belonging to the subsection.

## Additional Information

**How to cite this article**: Rodi, G. C. *et al.* Optimal Learning Paths in Information Networks. *Sci. Rep.* **5**, 10286; doi: 10.1038/srep10286 (2015).

## Supplementary Material

Supplementary Information
