# Supplementary material for: Optimal Learning Paths in Information Networks
Source: Sci Rep. 2015 Jun 1;5:10286. doi: 10.1038/srep10286 (PMC4450758; doi:10.1038/srep10286)
Supplement: Supplementary Information [file srep10286-s1.pdf]

Optimal learning paths in information networks
Supplementary Information

G.C. Rodi[1,2], V. Loreto[3,2,4], V.D.P. Servedio[5,3], F. Tria[2]

[1] *Polytechnic University of Turin, Dept. of Mathematical Sciences, Corso Duca degli Abruzzi, 24, 10129 Turin, Italy*
[2] *Institute for Scientific Interchange (ISI), Via Alassio 11C, 10126 Turin, Italy*
[3] *Sapienza University of Rome, Physics Dept., Piazzale Aldo Moro 2, 00185 Rome, Italy*
[4] *SONY-Computer Science Lab (CSL), 5, Rue Amyot, 75005, Paris, France*
[5] *Institute for Complex Systems (ISC-CNR), Via dei Taurini 19, 00185 Roma, Italy*

1 Tuning the learning rigidity

In the main paper, we define the function $F^*_{S_i}(\Delta_i t)$ as

$$F^*_{S_i}(\Delta_i t) = \frac{1}{2} \cdot \left\{ \tanh\left[\frac{LR}{b_{S_i(t)}}\left(\Delta_i t - \frac{b_{S_i(t)}}{2}\right)\right] + 1 \right\}. \quad \text{(S1)}$$

The free parameter LR (which stands for *learning rigidity*) allows us to control the slope of the function $F^i_{S_i}(\Delta_i t)$ in the interval $[0, b_{S_i(t)}]$, as reported in Fig. S1(a), for $b_{S_i(t)} = 2^{S_i(t)+3}$. How its value affects the learning efficiency is here tested and reported for the case of uncorrelated items, where the knowledge strength of each item $S_i(t)$ corresponds only to the number of reviews occurred up to time t.
While tuning the slope, the average number of units forgotten and the average number of time steps a unit has to wait in the queue before being reintroduced are reported in Fig. S1(b). In Fig. S1(c) we show how the change in the slope affects the introduction rate $n(t)$, and the exponent exp of the fitting function $n(t) \propto t^{exp}$. The data reported can be compared with the limit case of a step function for $F_{S_i}(t)$, which corresponds to the case $LR = \infty$. It is worth noting that an efficiency criterion based on the only introduction rate does not lead to the same evaluation of the learning performances as when the forgetting dynamics is considered. Indeed, while LR gets larger, the introduction rate monotonically increases, this corresponding to a faster learning process. Nevertheless, more units are forgotten during the procedure as well as more time is needed for them to be reintroduced from the forgetting queue.

2 Discussions on bounds $\mathbf{a_{S_i(t)}}$, $\mathbf{b_{S_i(t)}}$

In this section we compare results obtained on UCM[1] graphs with $\gamma = 2$ and order $N = 10^4$ when a polynomial bound $b_{S_i(t)} = S_i(t)^2$ is used instead of the exponential one $b_{S_i(t)} = 2^{S_i(t)+3}$ considered so far. Agendas are simulated on networks with increasing average connectivity and by using both the RL and the RS criterion for the new entry selection. Results are shown in Fig. S2.
In the first row, we show some introduction rates $n(t)$, which result to be slower than in the exponential case, as expected. Moreover, when the temporal window $[0, b_{S_i(t)}]$ useful for the $(k+1)$-th repetition is narrower, the order according to which the items are introduced becomes more relevant. To show this, in the second row we report the number of distinct units as a function of the relative difference between the time needed to introduce them using the RL or the RS criterion, namely $(t_{RL} - t_{RS})/t_{RL}$. Looking at the data two main results emerge. First, the difference in times is always positive, thus revealing that introducing the items randomly is less efficient than if the RS criterion is used. Secondly, how much the random criteria is less efficient depends more on the graph connectivity pattern if the bound enlarges polynomially rather than exponentially. In particular, for graphs with a more heterogeneous connectivity

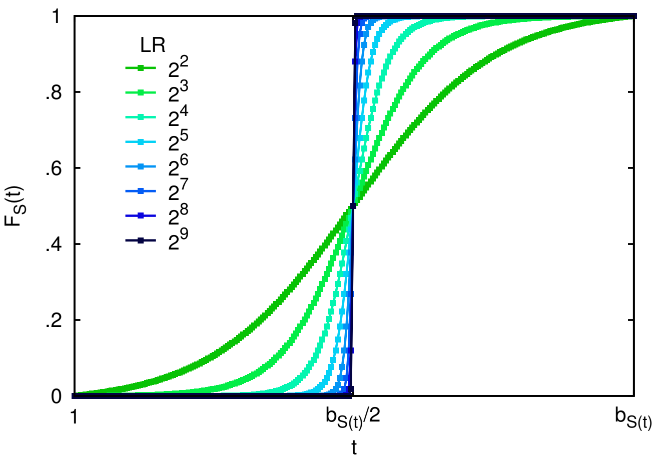

(a)

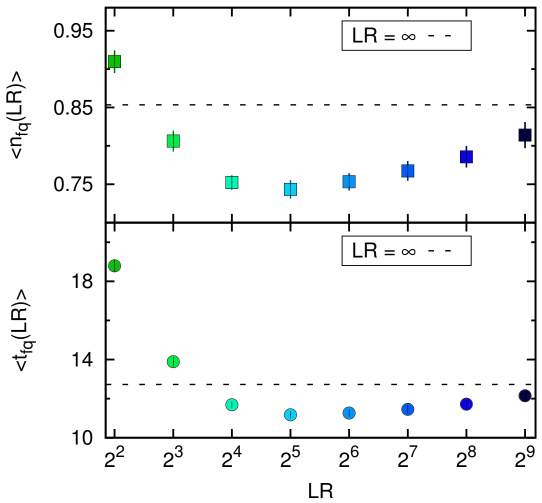

(b)

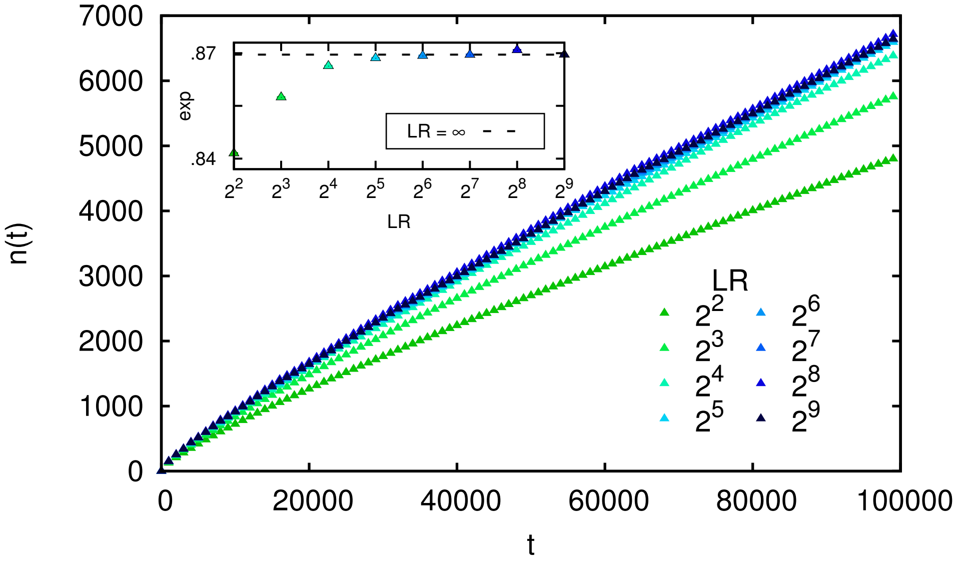

(c)

Supplementary Figure S1: **Learning rigidity and schedule efficiency.** In (a), we report different plots for the function $F_{S_i}(t)$ (Eq. S1) in the interval $[0, S_i(t)]$ while tuning the value of the parameter LR. Correspondingly, in the figures (b) and (c) we show some properties of the agendas obtained by running the simulations on sets of 10^4 disconnected nodes. In particular, in (b) we report the average number of units in the forgetting queue (subfigure at the top) and the average number of time steps a forgotten unit has to wait before being reintroduced (bottom). In (c) the introduction rates $n(t)$ are reported, with the exponent of the fitting function $n(t) \propto t^{exp}$ in the inset. All the data are averaged on 50 runs. Standard errors are reported.

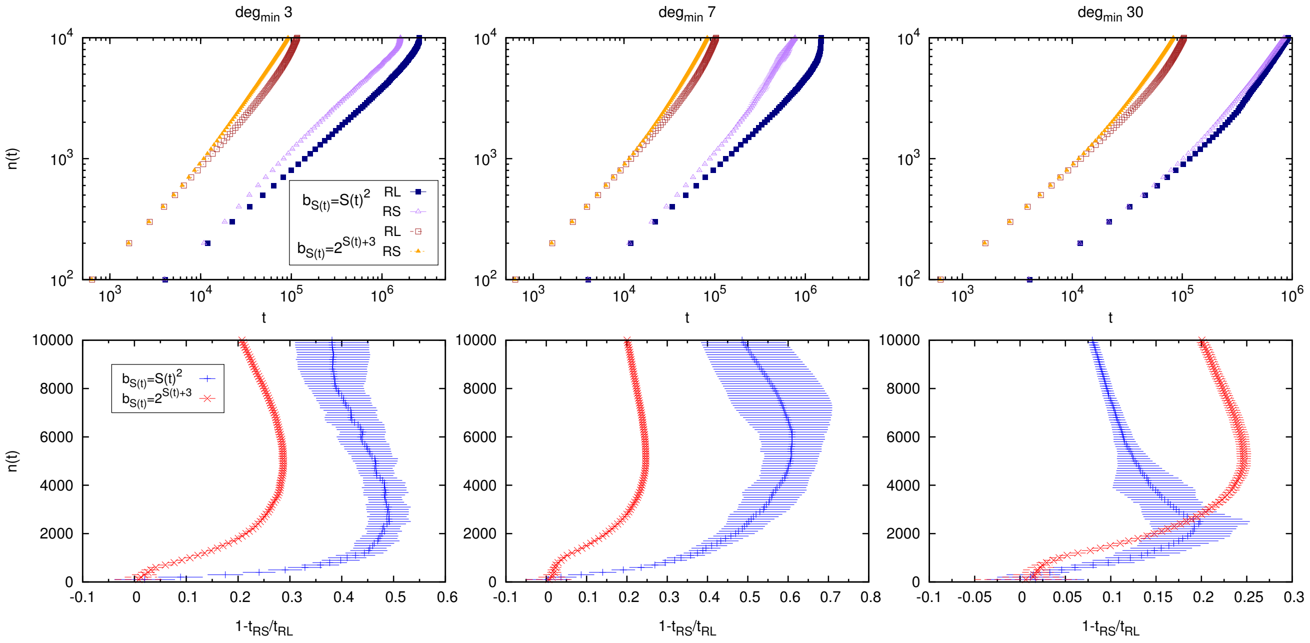

Supplementary Figure S2: **Comparison between polynomial and exponential bounds for the repetition time window width.** Comparison between the $b_{S_i(t)} = S_i(t)^2$ and $b_{S_i(t)} = 2^{S_i(t)+3}$ functional forms of the repetition time window upper bound $[0, b_{S_i(t)}]$. The schedules are simulated on UCM[1] graphs with $\gamma = 2$, order $N = 10^4$ and different minimum degree. Along the first row, the introduction rates $n(t)$ are shown as function of the time in the two cases when the RL or the RS criterion is used. Correspondingly, in the plots in the second row we report the number of units as a function of the relative difference in the average times needed to introduce them when the two criteria are used, namely $(t_{RL} - t_{RS})/t_{RL}$. Each agenda has been averaged over 50 runs. Standard errors are reported.

pattern, like the ones for which data are reported with minimum degree 3 or 7, the RS criterion allows to select the nodes more properly, thus resulting in a greater gain in efficiency if the repetition time window widths are reduced.

3 Tests on the active effect

In order to better analyse the role of the active effect on the learning efficiency, here we generate schedules in which each node enters the agenda with a pre-assigned k_0^i value, in this way keeping fixed and independent of the dynamics the total knowledge reinforcement gained throughout the procedure. In particular, for both the cases without passive effect and with a passive contribution ($\alpha = 0.1$):

- we generate a learning schedule on a optimal UCM[1] graph (we set the $degree_{MIN} = 7$ and $\gamma = 2$ in $P(deg) \propto deg^{-\gamma}$), using the RS entry selection criterion;
- we reshuffle on the entire graph the k_0^i with which each node was introduced in the previous agenda. Then we generate a new schedule on the same graph, without recomputing the active effect, rather using the pre-assigned values. The resulting agenda is referred to with *UCM resh.*;
- as in the previous step, we generate a further schedule starting from the same preassigned k_0^i but now considering as underlying an ER[2] graph, with average degree around 500 (this value corre-

sponding to a random graph yielding to the best performance, as reported in Figure 3 of the main paper).

In this way, while fixing the absolute values of the knowledge strength entering throughout the schedule because of the active effect, we destroy the correlation of the individual values k_0^i from the underlying graph.

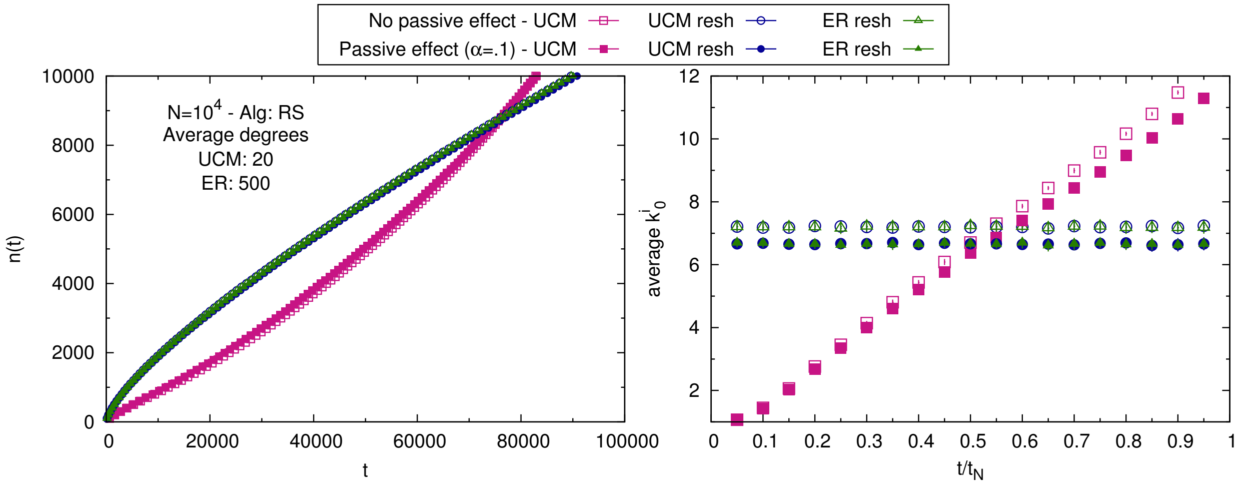

Supplementary Figure S3: **Results of the k_0^i reshuffling procedure.** Two series of 50 learning agendas are generated on a UCM graph with average degree 20 and $\gamma = 2$ (UCM, pink) without passive effect (empty symbols) and with it, setting $\alpha = 0.1$ (filled symbols). At the end of each schedule creation, the values of k_0^i are reshuffled over the nodes. Then, new agendas are generated both considering the same underlying UCM graph (UCM resh, blue) and a ER graph (ER resh, green) with average degree 500. In both cases, the nodes enter the agenda with the preassigned k_0^i-s. Of the resulting data, we report the introduction rate $n(t)$ (on the left) and the average k_0^i of introduced nodes as a function of their introduction time (subfigure on the right). In all the cases, we use the RS criterion to select the new entries.

The results are reported in Fig. S3. Since, on average, in the reshuffled cases the nodes enter the agenda with a higher starting knowledge strength, the introduction rates is initially faster in these cases. However, the final coverage times are higher than in the schedules without reshuffling. Moreover, in the reshuffled cases, no meaningful difference appears if the underlying topology is a random graph rather than a scale-free one. That means that all the scale-free graph properties useful in enhancing the learning procedure are ineffective if the active effect is separated from the dynamics.

4 Repetition selection criteria

We introduce two slightly modified versions for two of the entry selection rules described in the main paper: the preferential acquisition (PA) and the random surfing (RS). While using the same rules to select the nodes to be introduced, here we add a criterion based on the distance between nodes for selecting the item to be reviewed. Namely, say i to be the last node presented. Then, for every node j among the $n(t)$ already introduced, the quantity $F_{S_j}(\Delta_j t)$ is rescaled (before the normalizing procedure, Eq. (4) in the

main paper) as it follows:

$$F'_{S_j}(\Delta_j t) = \begin{cases} 0.9 \cdot F_{S_j}(\Delta_j t) & \text{if } j \in N_i \\ 0.1 \cdot F_{S_j}(\Delta_j t) & \text{otherwise} \end{cases} \tag{S2}$$

where we denote with N_i the neighbourhood of node i. With this rescaling, we obtain the new criteria RPA and RRS, with the new entries selected according to the PA or RS procedure respectively.

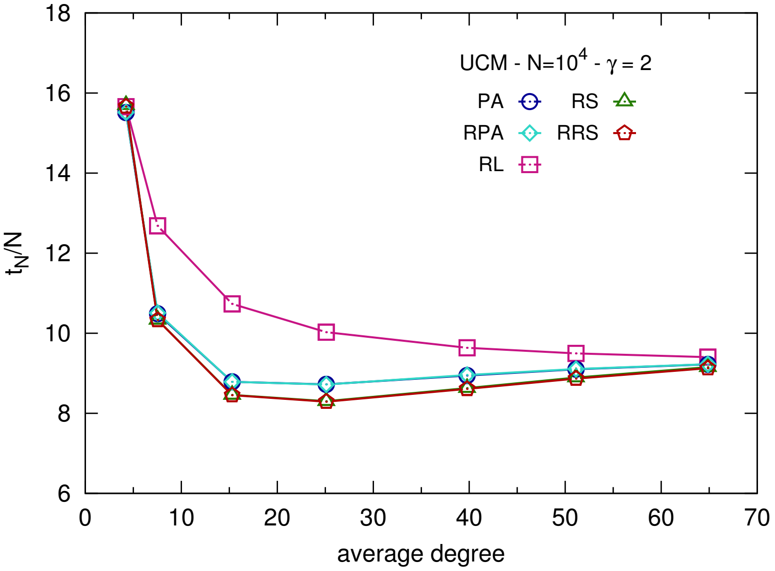

Supplementary Figure S4: **Comparison of graph exploration criteria.** We report the coverage times (scaled to the network order $N = 10^4$) obtained when the learning agendas are generated on UCM[1] graphs with $\gamma = 2$ and different values of the average degree. Different colors refers to distinct criteria for the selection of new nodes to be introduced (RS, PA, RL) or when a non uniform rule is used also to choose which unit has to be reviewed (RPA and RRS).

In Fig. S4 we report the coverage times resulting from simulations with the different criteria so far described (RL, PA, RS, RPA, RRS) on a UCM[1] graph with $N = 10^4$, $\gamma = 2$ and different average degrees, obtained by tuning the minimum degree allowed in the graph generation. The modification in the rule for the selection of the nodes to be repeated does not affect the resulting coverage times, as it emerges directly comparing the pairs PA-RPA and RS-RRS.

5 Miscellaneous results on synthetic graphs

5.1 Effects from graph finite size

A brief analysis of possible finite size effects on the learning efficiency is here reported on the two meaningful cases of ER and BA graphs with varying network order N.
For what concerns the passive effect, we still set $\alpha = 0.1$. Among the three entry selection criteria, only PA is used. In Fig. S5, it is reported the coverage time rescaled to each graph order N ($N = 5000,\ 10000,\ 20000$), as a function of the average connectivity. In both ER and BA cases, the same trends result while varying the number of nodes. To study the dependence of the coverage time on the graph order, the data have been re-elaborated, and the variation of the rescaled time with the order N

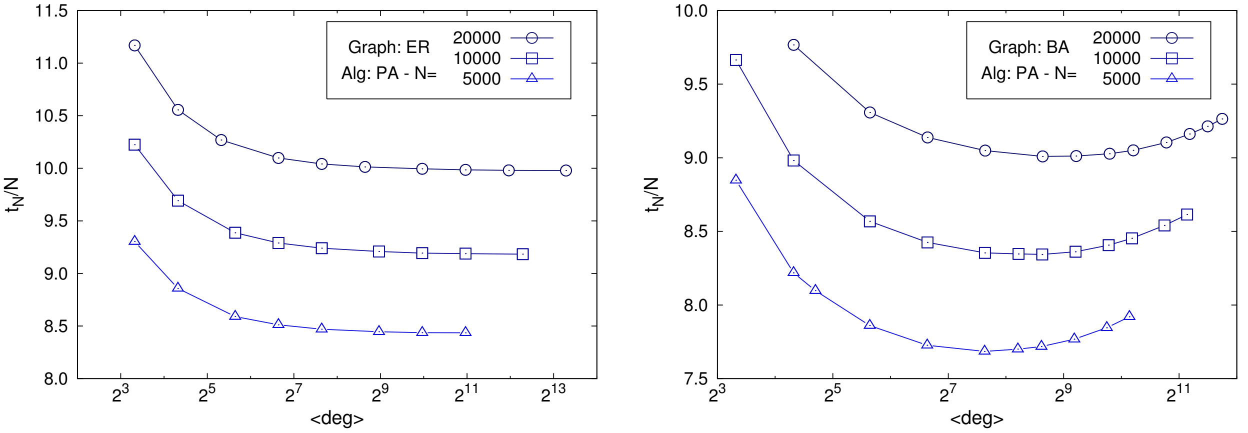

(a) (b)

Supplementary Figure S5: **Coverage time as a function of the network average connectivity for different network orders.** Data resulting from the agenda simulations on increasingly connected (a) random graphs (ER) and (b) scale-free networks, based on the Barabási-Albert model (BA), for three network orders: $N = 5000$ (triangles), 10000 (squares) and 20000 (circles). For each order, the coverage times have been rescaled to the number of nodes N. The data are averaged over 10 different graph realizations for each average connectivity, and 5 learning agendas for each of them. Standard errors are also reported, though they are not visible at this scale. The entry selection criterion is the preferential acquisition (PA). The passive effect is considered by setting $\alpha = 0.1$. The x axis is in log scale base 2.

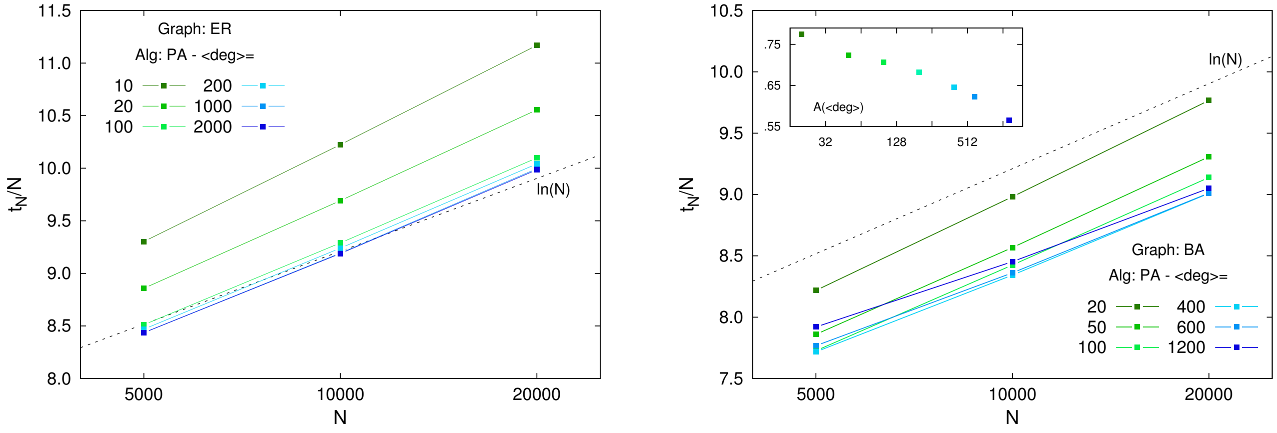

(a) (b)

Supplementary Figure S6: **Coverage time as a function of the network order N.** Elaboration of the data shown in Fig. S5. For different fixed values of the average degree $\langle deg \rangle$, the rescaled coverage time t_N/N is reported as a function of the network order N for (a) random graphs (ER) and (b) Barabási-Albert networks. The x axis is shown in logarithmic scale. In (b), for each average connectivity shown, a linear fit has been performed and the resulting fitted slope $A(\langle deg \rangle)$ is reported in the inset. In both the figures, a pure logarithmic function of N is reported with dashed black line.

has been considered, for some fixed values of the average connectivity. The results are shown in the subfigures S6(a) and S6(b). In the former, the data concerning the random graph are shown. Over the whole range of connectivity considered, a linear dependence of the scaled coverage time on $\log_2(N)$

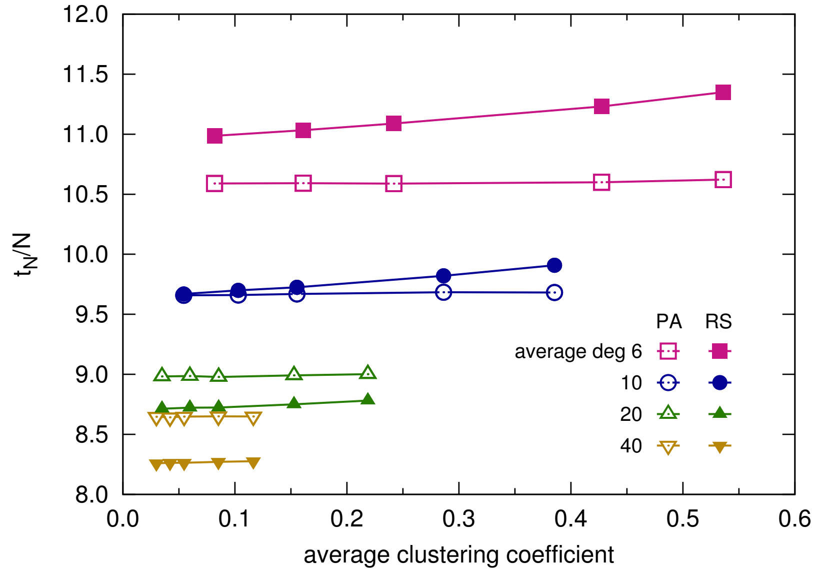

Supplementary Figure S7: **Coverage times and graph transitivity.** In the figure, we report the average coverage times scaled to the network order ($N = 10^4$) obtained on scale-free graphs generated according to the model proposed by Holme and Kim[3] as functions of the average clustering coefficient. Different colors refer to graphs with different minimum (and thus also average) degree, while we distinguish with empty or filled symbols data obtained when respectively the PA or the RS entry selection criterion is used.

seems to emerge, with the slope only slightly varying with the average degree $\langle deg \rangle$. Thus we conjecture

$$t_N(N) \propto N \log N. \tag{S3}$$

A similar behaviour of the dependence on N is valid also for the scale-free graph, as shown in Fig. S6(b). The slope of this linear trend varies now with the connectivity. Indicated with $A(\langle deg \rangle)$, it has been computed and reported in the inset.

5.2 The role of transitivity

In order to investigate the role of transitivity in determining the learning efficiency, simulations are conducted on graphs generated according to the model proposed by Holme and Kim[3]. So doing, while preserving the scale-free degree distribution, we can control the average clustering coefficient by properly tuning a parameter in the graph generation. Results for the coverage times on graph with different minimum degree are reported in Fig. S7, for both the PA and RS entry selection criteria. Changes in the transitivity do not affect the learning procedure when PA criterion is used, while a higher clustering coefficient in the network hinders the learning procedure if the RS rule is implemented.

5.3 On the passive effect and the role of hubs

How much the passive effect could influence the properties of the learning agendas is here discussed, considering an underlying scale-free topology generated through the UC model[1], with different proportion of hubs, namely with degree distribution $P(deg) \propto deg^{-\gamma}$, and $\gamma = \{2.0, 2.5, 3.0\}$. In particular, for the usual fixed order $N = 10^4$ and for the only entry selection criteria PA and RS, the agendas have been

simulated with a reduced passive effect, $\alpha = 0.05$, and without it, $\alpha = 0$. The resulting coverage times, together with the ones regarding the case $\alpha = 0.1$ are reported in Fig. S8.

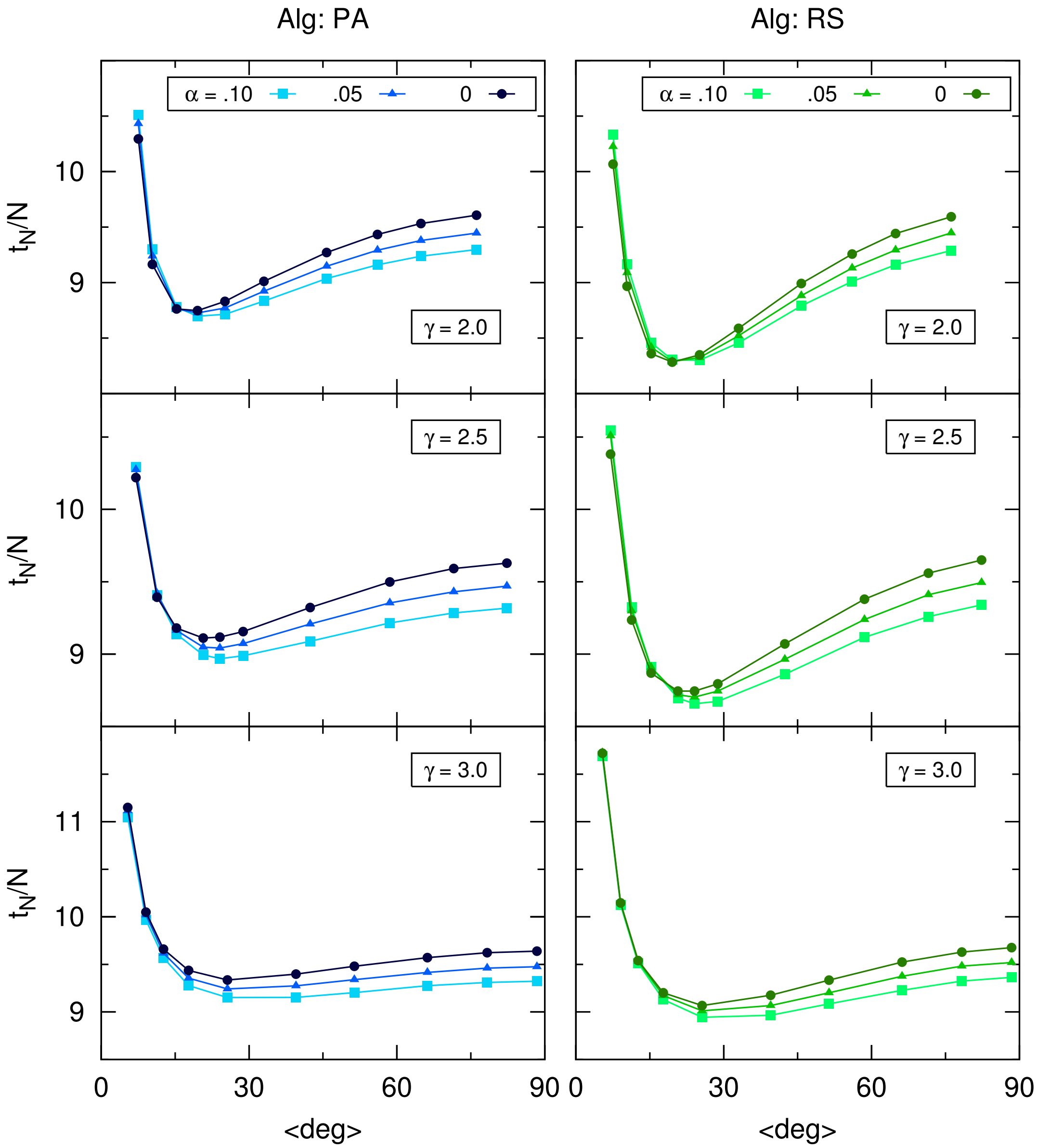

Supplementary Figure S8: **Passive effect and role of hubs in affecting the coverage times.** We report the coverage times scaled to the network order ($N = 10^4$) as a function of the average degree $\langle deg \rangle$. From the top, the sub-figures show data concerning UCM graphs with increasing exponent γ of the degree distribution $P(deg) \propto deg^{-\gamma}$ ($\gamma = 2.0, 2.5$ and 3.0). On the left the data are reported for agendas built with the entry selection criterion PA (main color blue) while on the right with RS (main color green). In each sub-figure, different symbols and main color variations refer to a different passive effect: $\alpha = 0.10$ squares and light main color, $\alpha = 0.05$ triangles and main color, $\alpha = 0$ dark main coloured circles. All the data are averaged over 10 graph realizations and, for each of them, over 5 agenda simulations.

It is worth pointing out two results. First, a reduction in the passive effect does not affect the coverage time uniformly over the range of connectivities. When different values of α are used, the spread in the

resulting coverage times is larger for highly connected graphs, regardless of the exponent γ of the degree distribution. This can be explained as follows. With high connectivity, the passive effect affects in a quite uniform way the entire network, i.e. the higher α, the longer the delay in the need of repetitions, and thus the minor the coverage time. A second observation regards the results in case of low average degree. In this cases, the degree distribution plays a role in determining the learning efficiency. In fact, while a reduction of the passive effect parameter α still leads to a (slightly) increase in the coverage time for $\gamma = 3.0$, the same reduction enhances the learning when $\gamma = 2.0$. A no-null passive effect, delaying the repetitions, could indeed lead to a reduction in the k_0^i of the new entries, if they are low-degree nodes. This can explain the differences between different exponents, since for the same average degree, the smallest the exponent γ, the smallest the allowed deg_{min} in the network.

6 Results on real graphs

6.1 Wikipedia subsections

We present here the results of simulations on both deeper cores for the Physics Wikipedia subsection and other sections, namely the Maths and Chemistry ones. The subgraphs were extracted as explained in the Methods section of the main paper. We refer to the same section also for a description of the perturbation procedures implemented.
To completion of the data presented in the main paper (Figure 4), in Fig. S9 we report the degree distribution for the original graph designed from the Physics section, its inner cores and their versions when extremely perturbed ($\pm 50\%$ of original links). Correspondingly, in Fig. S10 we show some results for the coverage times obtained on 5-, 7- and 10-core. As pointed out in the paper, the analysis of consecutive cores, together with the effects of the perturbation procedure, allow us to focus on the role of the least connected nodes in influencing the learning performance. Indeed, an optimal structure emerges with respect to further perturbations if the PA or RS criterion are used. This happens both when the cores beyond the second are considered and when the connectivity in the original structure is modified by inserting novel links, the percentage of links needed to reach a minimum depending on the procedure implemented for the perturbation. In both the cases, the hubs are only slightly modified, while the leaves are cut in the inner cores or reduced if the topology is positive perturbed.
Similar results emerge when considering the Maths or Chemistry sections. In Fig. S11 and S12 we report the degree distributions for the unperturbed original and inner cores graphs (red), and for their perturbed versions obtained when 50% of original links are removed (blue squares), added randomly (empty green triangles) or between second-neighbours (filled green triangles). The corresponding coverage times are shown in Fig. S13 and S14.
Also in these cases, the structures of the third cores lead to optimal schedules, with respect to reduction or increase of their connectivities. Furthermore, we notice again that is not the average degree the relevant quantity in determining the learning efficiency.

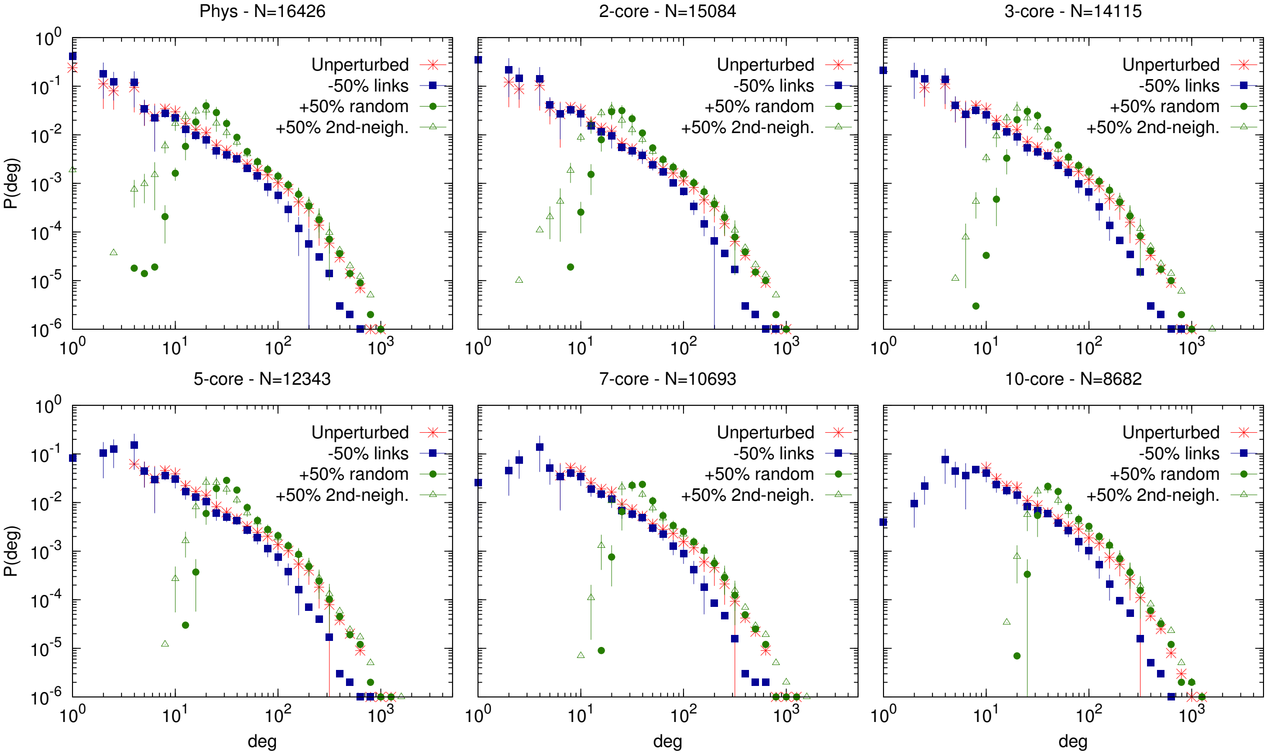

Supplementary Figure S9: **Degree distribution for original and perturbed Physics subgraphs.** Red symbols refer to the unperturbed graph and its inner cores. Starting from each unperturbed structure, 50% of links were randomly removed (blue squares), randomly added (filled green circles) or added only between second neighbors (empty green triangles). Both for the original graph and its cores, for each perturbation procedure 10 different perturbed graphs were generated. Standard deviations of data are shown.

6.2 Analysis and further results on HBC

For a deeper comprehension of the results obtained on the HBC graph and presented in the main paper (Figure 4), we report here the effects of the perturbation procedure on the strength and degree distribution. They are shown in Fig. S15 for the extreme case of $\pm 50\%$ of original links added or removed. We can notice that the original structure presents already an inferior cut-off in the node strengths. As further links are removed or added, whatever the procedure implemented, more time steps are needed to cover the network (as usual, if the PA or RS criterion is used).

Similar results are obtained if the graph is considered unweighted, i.e. each link is assigned with weight 1. The coverage times obtained in this case are shown in Fig. S16. Even if nodes exist in the original unperturbed graph with low degree, their presence is well-balanced, any perturbation making the learning procedure less efficient. Finally, by directly comparing these results with the coverage times obtained taking into account the link weights, it is found that the procedure is slightly more efficient in the last case, provided that the criterion used for the entry selection is the PA or the RS one.

6.3 EAT

In addition to the HBC graph, we analyse here the learning schedules obtained from a different graph of free associated words, namely the *Edinburgh Associative Thesaurus*[4,5]. It is a set of empirical word

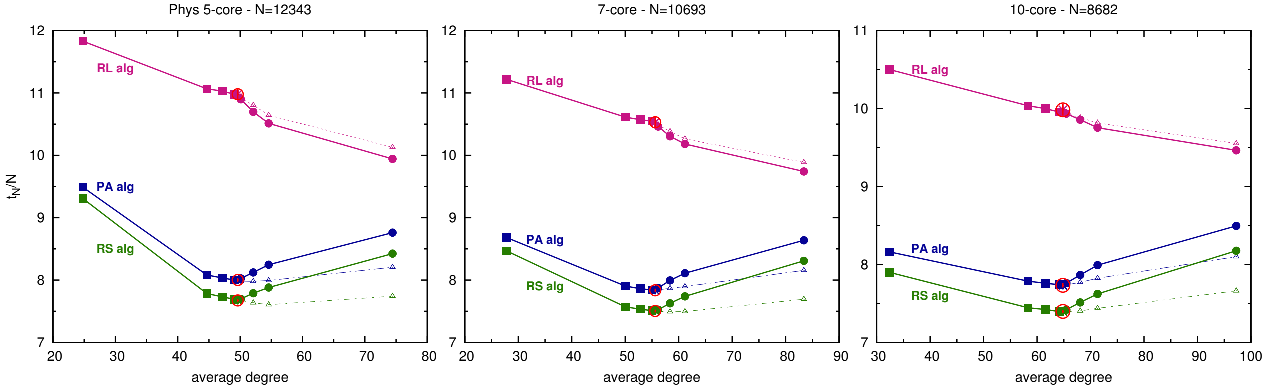

Supplementary Figure S10: **Coverage times on inner cores of Physics Wikipedia subsection.** We report the coverage time obtained on the unperturbed 5-, 7- and 10-cores of the Physics Wikipedia subsection (red circled data) and on some perturbed versions derived. In each figure, the squares refer to data resulting on graphs with reduced connectivity, obtained by randomly selecting and deleting different amounts of links in the original graphs. With circles and triangles data are reported when two different procedures for increasing the connectivity are considered. In the first case (circles, solid line), links are created by randomly selecting pairs of unconnected nodes. In the latter (triangles, dashed line), new links are added only between second-neighbour nodes. In all the cases, the fraction of links deleted/created are equal to 0.01, 0.05, 0.1 and 0.5. Different colors refer to the three criteria used to select the entries: random learning (RL, magenta), preferential acquisition (PA, blue) and random surfing (RS, green). The data are averaged over 50 agendas for the original graphs, while the other averages have been performed over 10 realizations of each perturbed graph and 5 agendas for each of them. Standard errors are reported.

association norms as they result from some discrete associative tasks conducted in a controlled experimental environment. Differently from the procedure implemented for the data collection in HBC, in this case either the number of participant, the starting bundle of cue words and the number of individuals who received a particular stimulus were controlled. A detailed description of the data collection method can be found in Kiss et al.[5].

For our aim, the undirected version of the original resulting network of word associations was considered, without self-loops. It resulted in a graph with 23,219 nodes and 289,116 associations.

The strength and degree distributions of the unperturbed graph are reported in Fig. S17, together with the ones obtained after having removed or added 50% of links. It can be noticed here the more significant presence of poor connected nodes with respect to the HBC graph. This results in a no efficient learning performance, as shown in Fig. S18. The structure can lead to faster schedules, if 10% of original links are randomly added, or even more if new connections are created between second neighbours or if the graph is treated as unweighted. With respect to this last case, as in HBC, taking into account the link weights in our schedule generation model allow us to (slightly) reduce the coverage times.

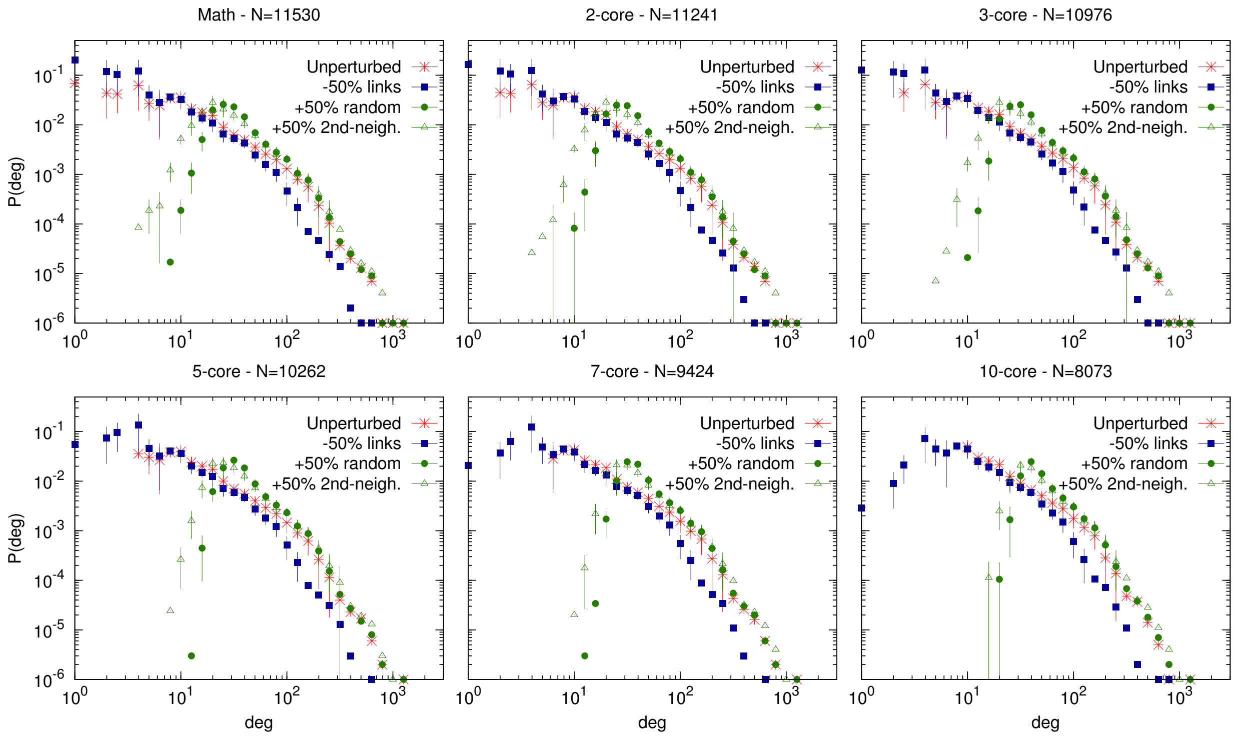

Supplementary Figure S11: **Degree distributions for original, and perturbed Maths subgraphs.** As for the Physics subgraph, Fig. S9.

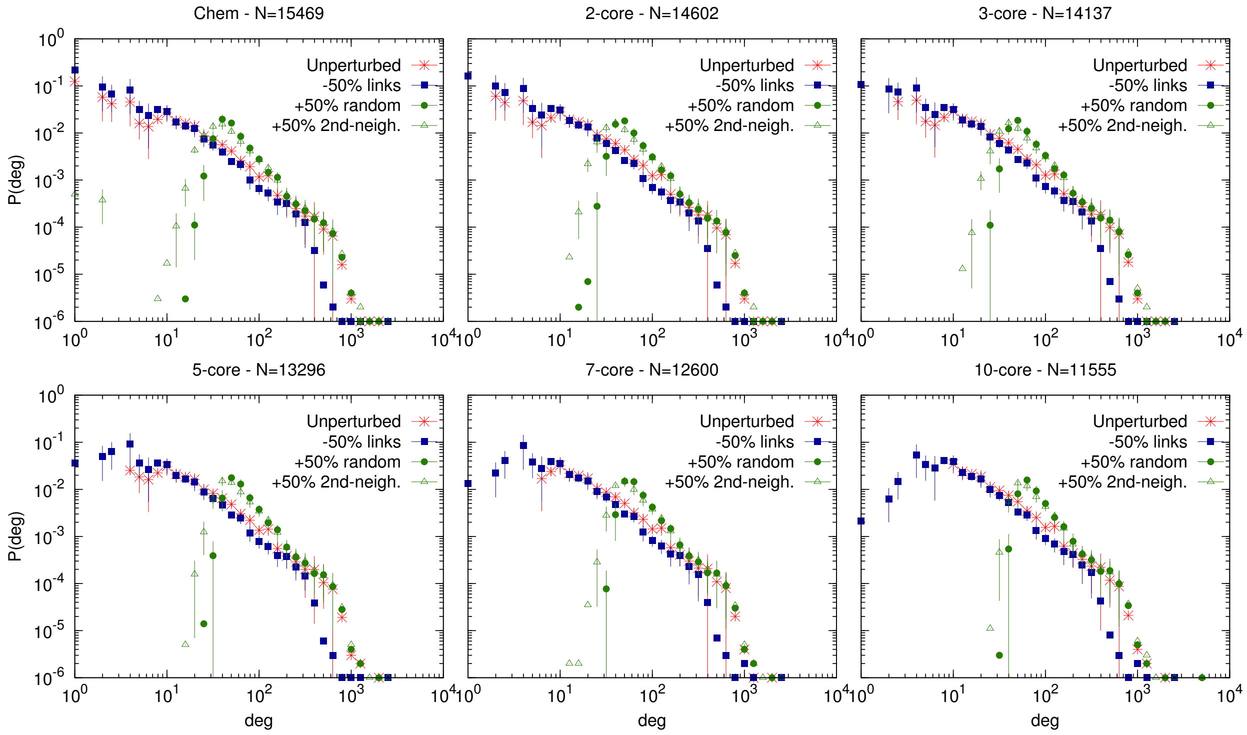

Supplementary Figure S12: **Degree distributions for original and perturbed Chemistry subgraphs.** As for the Physics subgraph, Fig. S9.

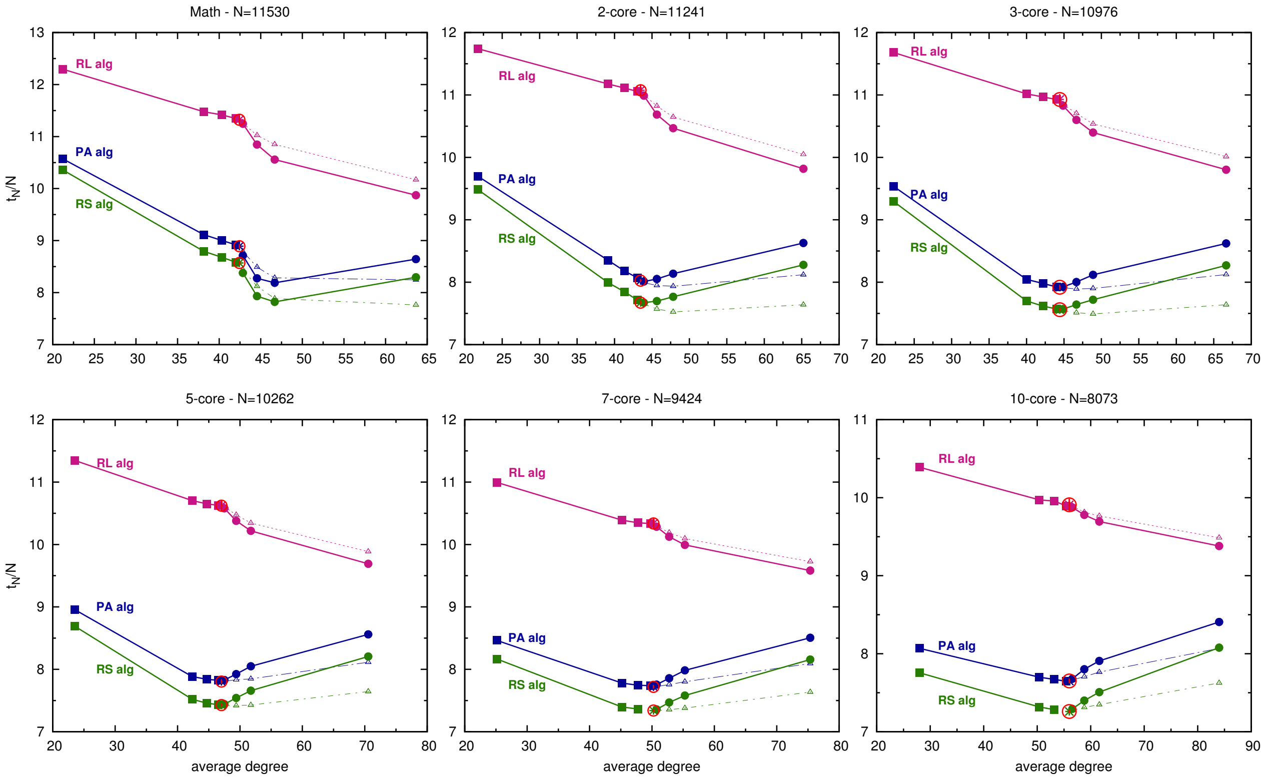

Supplementary Figure S13: **Coverage times on Maths Wikipedia subsection and its inner cores.** As in Fig. S10, we report the coverage time for the original Maths subsection and for different inner cores. In each subfigure, the red circled data refer to the agenda simulated on the unperturbed graph. Perturbation were performed deleting/creating the following fraction of links: 0.01, 0.05, 0.1 and 0.5. In particular, links were randomly deleted (squares), randomly added (circles) or added only between second neighbours (empty triangles). Different colors are used to distinguish among the entry selection criteria used: random learning (RL, magenta), preferential acquisition (PA, blue) and random surfing (RS, green). The data are averaged over 50 agendas for the original graphs, while the other averages have been performed over 10 realizations of each perturbed graph and 5 agendas for each of them. Standard errors are reported.

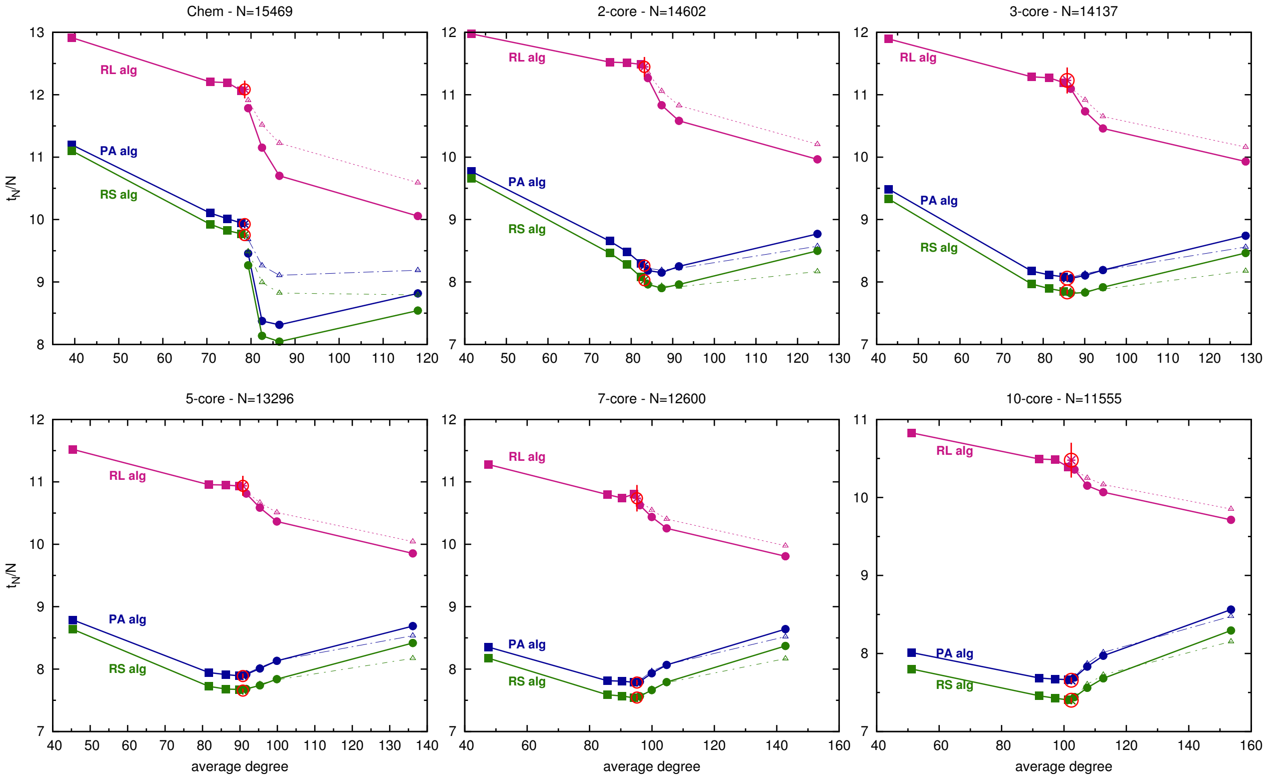

Supplementary Figure S14: **Coverage times on Chemistry Wikipedia subsection and its inner cores.** In each subfigure, the red circled data refer to the agenda simulated on the unperturbed graphs. Starting from these, perturbation were performed deleting/creating the following fraction of links: 0.01, 0.05, 0.1 and 0.5. In particular, links were randomly deleted (squares), randomly added (circles) or added only between second neighbours (empty triangles). With different colors we distinguish among the entry selection criteria used: random learning (RL, magenta), preferential acquisition (PA, blue) and random surfing (RS, green). The data are averaged over 50 agendas for the original graphs, while the other averages have been performed over 10 realizations of each perturbed graph and 5 agendas for each of them. Standard errors are reported.

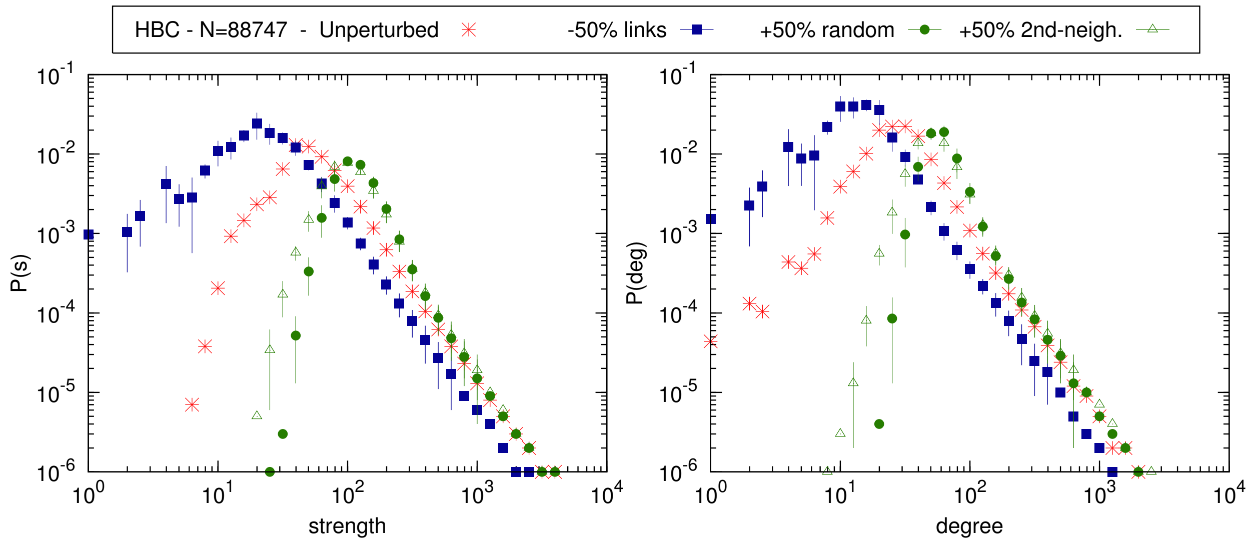

Supplementary Figure S15: **Strength and degree distribution for original and perturbed HBC graph.** Red symbols refer to the unperturbed graph. Starting from it, 50% of links were randomly removed (blue squares), randomly added (filled green circles) or added only between second neighbours (empty green triangles). For each perturbation procedure 10 different perturbed graphs were generated: data have been averaged on them. The resulting standard deviations are shown.

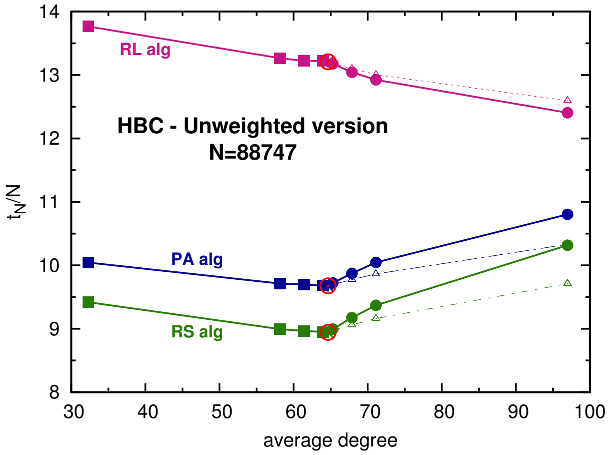

Supplementary Figure S16: **Coverage times obtained on unweighted HBC graph as a function of the average degree.** The red circled data refer to the agenda simulated on the unperturbed graph. From it, perturbation were performed deleting/creating the following fraction of links: 0.01, 0.05, 0.1 and 0.5. In particular, links were randomly deleted (squares), randomly added (circles) or added only between second neighbours (empty triangles). With different colors we distinguish among the entry selection criteria used: random learning (RL, magenta), preferential acquisition (PA, blue) and random surfing (RS, green). The data are averaged over 50 agendas for the original graphs, while the other averages have been performed over 10 realizations of each perturbed graph and 5 agendas for each of them. Standard errors are reported.

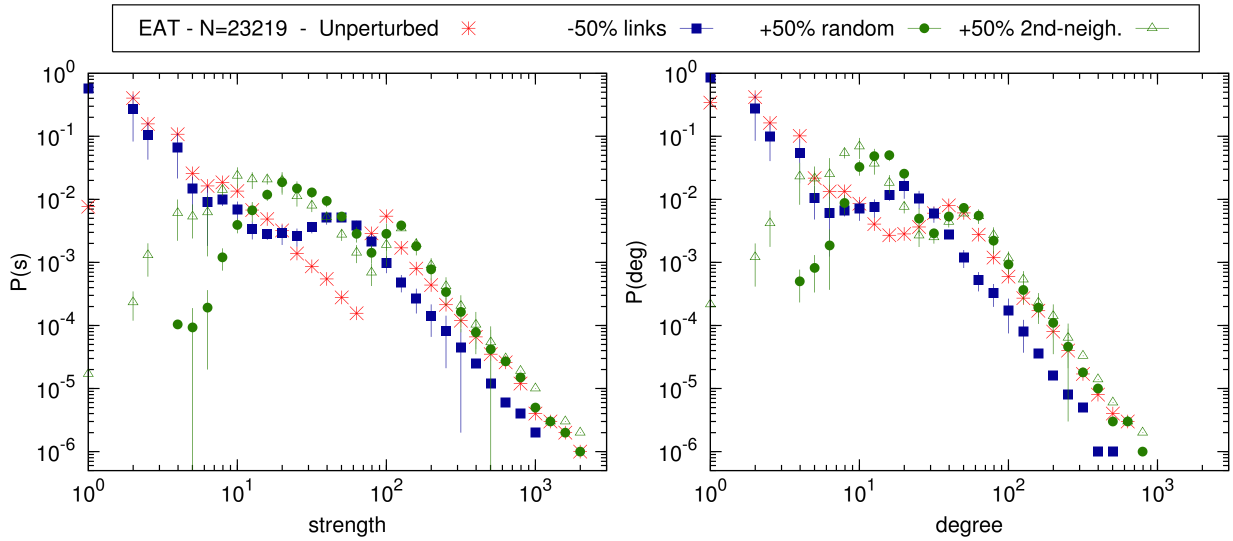

Supplementary Figure S17: **Strength and degree distribution for original and perturbed EAT graph.** As in Fig. S15, data referring to the unperturbed EAT graph are reported in red. From the original structure, 50% of links were randomly removed (blue squares), randomly added (filled green circles) or added only between second neighbours (empty green triangles). For each perturbation procedure 10 different perturbed graphs were generated and data have been averaged on them. The resulting standard deviations are shown.

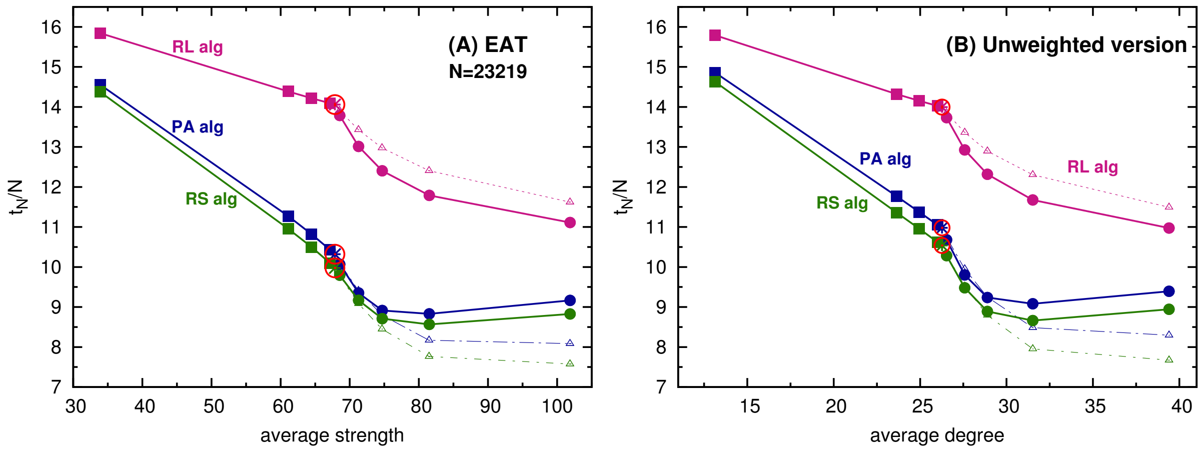

Supplementary Figure S18: **Coverage times obtained on weighted and unweighted perturbed EAT graphs.** The red circled data refer to the agenda simulated on the unperturbed graph, considered with the original weigths (on the left) or as unweighted (on the right). In both cases, perturbation were performed deleting or removing the following fraction of links: ± 0.01, ± 0.05, ± 0.1, $+0.2$, ± 0.5. In particular, links were randomly deleted (squares), randomly added (circles) or added only between second neighbours (empty triangles). In case of weighted graph, a weigh is assigned to each new link copying the weigh of a link among the original ones. With different colors we distinguish among the entry selection criteria used: random learning (RL, magenta), preferential acquisition (PA, blue) and random surfing (RS, green). The data are averaged over 50 agendas for the original graphs, while the other averages have been performed over 10 realizations of each perturbed graph and 5 agendas for each of them. Standard errors are reported.

References

1. Catanzaro, M. & Pastor-Satorras, R. Generation of uncorrelated random scale-free networks. *Phys. Rev. E* **71**, 4 (2005). `0408110v1`.

2. Erdös, P. & Rényi, A. On the evolution of random graphs. *Publ. Math. Inst. Hung. Acad. Sci.* **5**, 17–61 (1960).

3. Holme, P. & Kim, B. Growing scale-free networks with tunable clustering. *Phys. Rev. E* 2–5 (2002). `0110452v1`.

4. *Edinburgh Associative Thesaurus* website. `http://www.eat.rl.ac.uk/`. Date of access: 17/05/2013.

5. Kiss, G. R., Armstrong, C., Milroy, R. & Piper, J. An associative thesaurus of English and its computer analysis. In Aitkin, A. J., Bailey, R. W. & Hamilton-Smith, N. (eds.) *The computer and literary studies* (University Press, Edinburgh, 1973).
